# The relationship between workplace risk assessments and measures to manage psychosocial risks at work: findings from ESENER

**DOI:** 10.1007/s00420-025-02158-3

**Published:** 2025-07-31

**Authors:** David Beck, Morten Wahrendorf, Sabine Sommer, Mariann Rigó, Uwe Lenhardt, Thorsten Lunau

**Affiliations:** 1 Federal Institute for Occupational Safety and Health (BAuA), Nöldnerstr. 40-42, 10317 Berlin, Germany; 2 Medical Faculty, Institute of Medical Sociology, Centre for Health and Society, University of Duesseldorf, Duesseldorf, Germany; 3 Institute for Social Research and Social Economy (iso), Saarbruecken, Germany

**Keywords:** Occupational safety and health, Workplace risk assessment, Psychosocial risk management, Measures

## Abstract

**Purpose:**

Workplace risk assessment (WRA) is crucial for the management of psychosocial risks at work (PSRM), but some enterprises may also implement PSRM measures without formal WRA, in particular small and micro enterprises. This study analyses the associations between WRA and PRSM, and whether the associations vary by company size.

**Methods:**

The data come from the European Survey of Enterprises on New and Emerging Risks (ESENER) collected at the enterprise level in the EU-28 countries in 2014 (*n* = 40,584) and 2019 (*n* = 39,711). We distinguish eight measures of PSRM, and assess whether companies conduct a comprehensive WRA that includes psychosocial risks.

**Results:**

Many companies reported PSRM measures. The lowest rates were for “intervention in the case of long working hours” (2014: 26%, 2019: 32%), while the highest rates were for “procedures in the case of threats” (56%, 60%). Enterprises with a comprehensive WRA are more likely to implement measures (even after controlling for company size, industry, sector and country), but some enterprises have implemented PSRM even in the absence of a WRA, especially in 2014 or in smaller companies (5–49 employees). For example, findings suggest that in 2014 40% of the enterprises without a WRA have implemented “procedures in the case of threats” (2019: 46%).

**Conclusion:**

The findings underline the importance of a WRA that includes psychosocial risks as a means of implementing PSRM measures, but also advocates for a broader perspective that considers measures taken independently of legal occupational safety and health (OSH) standards, especially in small and micro enterprises.

## Introduction

Adverse psychosocial working conditions—e.g. job strain, effort reward imbalance, long/irregular working hours, or lack of support from supervisors or colleagues– contribute to the development of several widespread physical and mental diseases, such as coronary heart disease (Kivimäki et al. 2012; Dragano et al. 2017; Niedhammer et al. 2022) or depression (Theorell et al. 2015; Madsen et al. 2017; Seidler et al. 2022). In order to protect the health and safety of employees, it is therefore important that employers also take measures to reduce psychosocial risks at work such as considering the design of job content/tasks, work organisation, working time, social relations at work, the working environment and the use of work equipment (Leka and Cox 2008).

Available evidence suggests that measures to address psychosocial risks at work are far from being widely implemented (European Agency for Safety and Health at Work 2022, pp 54f): For example, findings of the European Surveys of Enterprises on New and Emerging Risks (ESENER) indicate that most enterprises in the member states of the European Union (EU-27) have not implemented any of the following measures: reorganisation of work in order to reduce work pressure, intervention in cases of excessively long or irregular working hours, or procedures for dealing with possible cases of bullying or threats. The findings also suggest that such measures are consistently more common in larger companies than in small companies. These findings highlight the need to improve the management of psychosocial risks in many places, particularly in small companies (< 50 employees).

According to the European Framework Directive on Safety and Health at Work (Council of the European Communities 1989), which is the basis for the occupational safety and health (OSH) legislation in the countries of the European Union (EU), the measures necessary to protect the safety and health of employees must be defined, implemented and checked for effectiveness as part of a workplace risk assessment (European Commission 1996). All sources of risk, including psychosocial factors, should be considered. Where psychosocial risks have been identified, measures must be taken to eliminate or minimise these risks as far as possible, and the effectiveness of the measures taken should be reviewed (European Agency for Safety and Health at Work 2014; Beck and Lenhardt 2019).

Against this background, the implementation of comprehensive workplace risk assessments (WRAs) that also take psychosocial risks into account can be seen as a precondition for the implementation of psychosocial risk management (PSRM) measures. Available research provides evidence that companies with a well-established OSH management system (which includes the implementation of WRA) often also have procedures and measures in place to manage psychosocial risks (European Agency for Safety and Health at Work 2012; Dahler-Larsen et al. 2020). PSRM should therefore be treated as an essential part of an overall OSH management system that is integrated into the organisation’s OSH policy and WRA process (Leka and Cox 2008). In this sense, PSRM ideally includes both workplace risk assessment, taking into account psychosocial risks, and measures to reduce these risks (PSRM = WRA + Measures). 

In recent years, a wide range of measures, including both legislative and non-legislative approaches, have been developed at the European and national levels by various stakeholders with the aim of improving the integration of psychosocial risk management into companies’ OSH policy and WRA (European Foundation for the Improvement of Living and Working Conditions and European Agency for Safety and Health at Work 2014; Leka et al. 2015). There are therefore good reasons to expect that the link between WRA and measures on psychosocial risks has become stronger over time. On the other hand, explorative field studies have also shown that targeted measures to reduce psychosocial risks are also taken independently of the implementation of WRAs and thus outside the structures and processes of (institutionalised) OSH management (Beck et al. 2017; Lenhardt 2017). Instead of referring to formal OSH requirements and standards, the implementation of measures may refer to the company’s culture and values or to the standards of the profession, standards of good leadership or standards of professional human resource management (Pinder et al. 2016; Beck et al. 2017). Accordingly, these field studies clearly indicate that contributions to the effective management of psychosocial risks are necessary and possible in multiple and diverse company contexts, where the job tasks, the work organisation, the working time, the social relations at work and the work environment are evaluated and (re)designed. Looking only at the implementation of WRAs therefore does not seem to be sufficient to understand whether and to what extent psychosocial risks are both addressed and managed in companies. This would be particular important for small enterprises, which often lack systematic OSH management and WRAs (Hasle and Limborg 2006; Masi and Cagno 2015; Walters and Wadsworth 2016).

Studies that examine the implementation of specific PSRM measures in the context of the presence or absence of WRAs are rare. Existing studies, such as the company survey for the evaluation of the Joint German Occupational Safety and Health Strategy, for example, examine the implementation of WRAs and their consideration of psychosocial factors (Beck and Lenhardt 2019). However, the specific measures that have been taken within or outside this context to minimize psychosocial risks at work have not been analysed. Overall, there is a lack of studies that shed equal light on both the consideration of psychosocial risks in WRAs and the implementation of concrete measures to reduce these risks. In particular, there is a lack of studies that examine the relationship between these two aspects of PSRM and allow far-reaching conclusions to be drawn about the extent to which specific risk reduction measures are implemented in the presence or the absence of comprehensive WRAs. The overarching aim of this study is to fill this knowledge gap by answering the following questions:

1. How does the proportion of companies in the member states of the European Union (EU-28, as of 2019) implementing PSRM measures vary with the presence or absence of a comprehensive WRA?
2. Is the extent of comprehensive WRAs associated with a higher likelihood of PSRM measures?
3. Has the association between the extent of comprehensive WRAs and the likelihood of implementing PSRM measures changed over time?
4. Is the association between the extent of comprehensive WRAs and the likelihood of implementing PSRM measures less pronounced for small enterprises than for large companies?

## Materials and methods

### Data sources

This study is based on data from the European Survey of Enterprises on New and Emerging Risks (ESENER), Survey wave 2 (European Agency for Safety and Health at Work 2016) and wave 3 (European Agency for Safety and Health at Work 2022). Both ESENER-2 and ESENER-3 were conducted by the European Agency of Occupational Safety and Health (EU-OSHA). ESENER-2 and 3 provide data on many aspects of the organisation of occupational safety and health (OSH), including the implementation of WRAs and measures to prevent psychosocial risks at work, and therefore provide a very good data base for investigating our questions. We consider both waves of ESENER in order to test our hypotheses on the association between WRA and measures on two independent samples, thus increasing the robustness and generalisability of the results. Secondly, it allows us to analyse and identify any changes over time.

ESENER-2, conducted in 2014, surveyed 49,320 establishments with five or more employees in 36 European countries on how health and safety, and in particular about how psychosocial risks are managed in their workplaces (European Agency for Safety and Health at Work 2016). ESENER-3, conducted in 2019, surveyed 45,420 establishments with five or more employees in 33 European countries (European Agency for Safety and Health at Work 2022). Within each surveyed establishment, the person who knows the most about health and safety in that establishment was interviewed by a computer assisted telephone interview (CATI). The fieldwork was conducted in accordance with generally accepted procedural standards, incorporating various measures to minimise self-selection bias, enhance response rates and included trained interviewers.

The ESENER samples were obtained through a multi-stratified random sampling procedure, employing a sampling matrix that differentiated between sectors and sizes. For the sampling by size (5–9, 10–49, 50–249, 250+), a deliberately disproportional sample design was chosen. The weighting factors available in the datasets include corrections for these design effects, as well as any potential selective non-response by sector or size. To assess the deviation of the weighted data from the unweighted data the effectiveness is reported. In view of the disproportionalities, average effectivity rates of nearly 60% in ESENER-2 and 70% in ESENER-3 are considered to be good values (European Agency for Safety and Health at Work 2015; European Agency for Safety and Health at Work 2019a). A more detailed description of the survey methodology (including the master questionnaire, details on sampling strategy, non-response rates and weights) can be found in the technical reports (European Agency for Safety and Health at Work 2015; European Agency for Safety and Health at Work 2019a).

For the analyses presented in this article, only data from the 28 member states of the European Union (as of 2019), which are covered by the European Framework Directive on Safety and Health at Work, were used (total *n* = 80,295 with 40,584 establishments in ESENER-2 and 39,711 in ESENER-3). This directive, which came into force in 1989, serves to harmonise and improve national OSH law and stipulates, among other things, that all EU member states must oblige companies in their country to define OSH measures based on workplace risk assessments, and to also take psychosocial risks into account when doing so (Council of the European Communities 1989).

ESENER 2014 and 2019 datasets can be accessed via the UK Data Service of the University of Essex and the GESIS Datenarchiv Köln (see data availability).

### Variables

#### Workplace risk assessment (WRA)

In the ESENER-2 and 3 several questions are related to the process of workplace risk assessment. Specifically, survey items address (1) whether WRAs are regularly carried out and (2) what aspects are evaluated in these workplace risk assessments. Aspects of the psychosocial working environment that were included in the ESENER questionnaire are “supervisor-employee relationships” and “organisational aspects such as work schedules, breaks or work shifts”. Based on the answers to these questions, the companies’ risk assessment practices were categorised for further analysis as follows:

- WRA A: Respondents did not confirm that WRAs were carried out in their company.
- WRA B: WRAs were confirmed, but no psychosocial aspect was considered.
- WRA C: Carrying out WRAs was confirmed and one out of two aspects of psychosocial risks was considered.
- WRA D: Carrying out WRAs was confirmed and two out of two aspects of psychosocial risks were considered. This value is used as an indicator of a comprehensive WRA.

#### Strategies and measures to prevent psychosocial risks (PSRM measures)

Beyond the question of risk assessment, ESENER-2 and −3 data are used to analyse different measures to prevent psychosocial risks. Such measures are covered by several questions. Specifically, respondents were asked to indicate (1) whether their establishments have an action plan to prevent work-related stress, and whether there are procedures in place to deal (2) with possible cases of bullying or harassment or (3) with possible cases of threats, abuse or assault by clients, patients, pupils or members of the public. These questions were only asked in companies with at least 20 employees. This rationale is based on the results of the cognitive pretest interviews, which indicated that a proportion of respondents from small organizations considered these questionnaire items irrelevant to their context due to the limited size of their workplaces (European Agency for Safety and Health at Work 2019b). They are also asked to report which of the following measures are used in their company to prevent psychosocial risks: (4) reorganisation of work in order to reduce job demands and work pressure, (5) confidential counselling for employees, (6) conflict resolution procedures, (7) intervening in cases of excessive long or irregular working hours. In addition, respondents indicate (8) whether employees are trained on how to prevent psychosocial risks such as stress or bullying. ESENER-3 also asked whether employees are allowed to make more decisions about their job task. We have not included this measure in the analyses as it is not available in ESENER-2.

### Statistical analyses

First, descriptive analyses of weighted data were performed to calculate the weighted proportion of establishments implementing the different measures described above to deal with psychosocial risks by categories of risk assessment, differentiated for survey years 2014 and 2019. Furthermore, logistic regression analyses for each dependent PSRM measure were performed to analyse the association between the implementation of WRA and measures to prevent psychosocial risks. For these analyses we used a combined dataset of the 2014 and 2019 data and present results separated for years. Furthermore, we calculated interaction effects to analyse whether there was a statistically significant difference in the association between the years. The dependent variables in each regression are the different types of measures to deal with psychosocial risks (in the form of dummy variables). The main independent variable indicates whether and what type of risk assessment is carried out. Additionally, control variables are included for the size of the company, branches, sector and country. The results are presented in the form of adjusted predicted percentages (PP) and average marginal effects (AME) (Mood 2010; Williams 2012). These estimates are more intuitive and easier to interpret than odds ratios. They can also be compared across different models, which is not possible when using odds ratios (Mood 2010). We calculate the PP of implementing different types of psychosocial risk measures for each category of WRA described above. PP are based on the “margins” command in Stata (sometimes also named “average adjusted predictions”), and quantify the predicted (i.e. adjusted) proportions of companies that have implemented the respective PSRM measure under study by categories of WRA. The AME is then easily calculated by taking the differences in the PPs. For example, if the PP for ”interventions in case of long working hours” is predicted to be 30% for companies without WRA (WRA-A) and 50% for companies with comprehensive WRA (WRA-D), then the AME (D-A) is 20, meaning that the proportion is predicted to be 20% points higher for companies with comprehensive WRA compared to companies with no WRA. To test if the association between the extent of comprehensive WRAs and the likelihood of implementing PSRM measures is less pronounced for small enterprises than for large companies, we also use the combined dataset of the 2014 and 2019 datasets and present data separated for company size. Second order interaction effects of having a comprehensive WRA depending on the size of the company were estimated as the difference between the AME of small and larger companies.

All computations were carried out using Stata 17.

## Results

### Sample description

Table 1 describes the sample of companies (unweighted numbers and percentages of observed enterprises) for the variables included in this study, differentiated for survey years 2014 and 2019. The samples are largely composed of small companies (< 50 employees), service sector companies, and private businesses. Both in 2014 and 2019, just over 80% of the companies observed in this sample confirmed that they had implemented WRAs, but less than half of these reported that both psychosocial factors were considered in the WRA (comprehensive WRA). The percentage of companies reporting measures to deal with psychosocial risks ranged 2014/2019 from 26.9%/31.2% (interventions in the case of long working hours) to 57.7%/63.3% (procedures to deal with possible threats). Information on the existence of an “action plan to prevent work-related stress” and “procedures to deal with possible cases of bullying or harassment” is only available for companies with 20 or more employees, i.e. only for half of the total sample. Information on the existence of “procedures to deal with possible cases of threats, abuse or assault by clients, patients, pupils or members of the public” is only available for companies with at least 20 employees and for those confirming that their employees have to deal with difficult customers, patients, pupils etc. in their work.

**Table 1:** Sample description: number of observations and percentages (unweighted)

		2014 (ESENER 2)	2019 (ESENER 3)
		*n* = 40,584	*n* = 39,711
		Obs. (%)	Obs. (%)
Workplace risk assessment	WRA-A	7,265 (18.1)	7,166 (18.3)
	WRA-B	8,255 (20.5)	6,932 (17.7)
	WRA-C	9,200 (22.9)	8,899 (22.8)
	WRA-D	15,503 (38,5)	16,105 (41,2)
Action plan stress^a^	No	14,264 (64.6)	10,437 (58.8)
	Yes	7,818 (35.4)	7,315 (41.2)
Procedure bullying^a^	No	10,600 (47.6)	7,477 (41.7)
	Yes	11,651 (52.4)	10,460 (58.3)
Procedure threats^b^	No	5,600 (42.3)	4,260 (36.7)
	Yes	7,652 (57.7)	7,333 (63.3)
Reorganisation	No	23,429 (58.9)	20,794 (53.7)
	Yes	16,330 (41.1)	17,894 (46.3)
Confidential counselling	No	22,986 (58.0)	20,903 (54.0)
	Yes	16,675 (42.0)	17,828 (46.0)
Conflict resolution procedure	No	26,319 (66.2)	22,529 (57.9)
	Yes	13,423 (33.8)	16,382 (42.1)
Intervention long working hours	No	29,006 (73.1)	26,295 (68.8)
	Yes	10,664 (26.9)	11,931 (31.2)
Training psychosocial risks	No	24,061 (60.2)	23,090 (59.2)
	Yes	15,935 (39.8)	15,891 (40.8)
Company size	< 50	27,645 (68.1)	29,198 (73.5)
	≥ 50	12,939 (31.9)	10,513 (26.5)
Industry	Agriculture, forestry and fishing - Manufacturing industry	8,219 (20.3)	6,152 (15.5)
	Mining - Energy supply - Water supply - Construction	3,941 (9.7)	3,620 (9.1)
	Trade - Transportation - Accomodation - Arts	11,716 (28.9)	12,746 (32.1)
	IT - Finances - Services	7,314 (18.0)	7,877 (19.8)
	Public Administration– Defence - Social Insurance	2,585 (6.4)	1,950 (4.9)
	Education/teaching - Health and social services	6,809 (16.8)	7,366 (18.5)
Sector	Public sector	8,226 (20.4)	8,397 (21.3)
	Private sector	32,178 (79.6)	31,096 (78.7)

A = no WRA; B = WRA without consideration of psychosocial risks; C = WRA considering one aspect of psychosocial risks; D = WRA considering two aspects of psychosocial risks

^a^ only companies with ≥ 20 employees

^b^ only companies with ≥ 20 employees and companies where employees have to deal with difficult customers, patients, pupils etc

### Proportion of enterprises with PSRM measures, by categories of WRA

Table 2 shows the proportion of companies (weighted percentages and absolute numbers of establishments observed) in 2014 and 2019 that have confirmed specific measures to deal with psychosocial risks, depending on workplace risk assessment.

**Table 2:** Proportion of companies implementing measures to deal with psychosocial risks by risk assessment category, by year

		Workplace Risk Assessment									
		2014 (ESENER 2)					2019 (ESENER 3)				
		WRA-A	WRA-B	WRA-C	WRA-D	Total	WRA-A	WRA-B	WRA-C	WRA-D	Total
		%(w)	%(w)	%(w)	%(w)	%(w)	%(w)	%(w)	%(w)	%(w)	%(w)
Action plan stress^a^	No	86.5	81.6	68.8	49.8	66.0	86.5	76.0	63.5	44.0	59.5
	Yes	13.5	18.4	31.2	50.2	34.0	13.5	24.0	36.5	56.0	40.5
	n(unw)	2,390	4,386	5,580	9,578	21,934	1,948	2,938	4,312	8,373	17,571
Procedure bullying ^a^	No	70.7	62.6	55.6	39.5	52.4	67.1	59.2	48.8	33.8	45.9
	Yes	29.3	37.4	44.4	60.5	47.6	32.9	40.8	51.2	66.2	54.1
	n(unw)	2,389	4,430	5,618	9,664	22,101	1,947	2,984	4,367	8,446	17,744
Procedure threats ^b^	No	61.6	56.0	48.4	31.3	44.3	55.8	56.7	44.6	28.6	40.4
	Yes	38.4	44.0	51.6	68.7	55.7	44.2	43.3	55.4	71.4	59.6
	n(unw)	1,523	2,325	3,243	6,065	13,156	1,336	1,744	2,796	5,589	11,465
Reorganisation	No	66.8	71.4	60.8	52.5	61.5	61.9	67.4	57.0	43.4	54.8
	Yes	33.2	28.6	39.2	47.5	38.5	38.1	32.6	43.0	56.6	45.2
	n(unw)	7,185	8,095	8,994	15,168	39,442	7,056	6,750	8,638	15,708	38,152
Confidential counselling	No	68.0	72.5	64.7	54.1	63.3	63.4	71.0	59.8	46.8	57.7
	Yes	32.0	27.5	35.3	45.9	36.7	36.6	29.0	40.2	53.2	42.3
	n(unw)	7,195	8,073	8,959	15,115	39,342	7,058	6,742	8,654	15,745	38,199
Conflict resolution procedure	No	77.6	78.9	72.1	59.6	70.3	77.1	75.8	64.7	49.5	63.6
	Yes	22.4	21.1	27.9	40.4	29.7	22.9	24.2	35.3	50.5	36.4
	n(unw)	7,201	8,083	8,990	15,142	39,416	7,087	6,782	8,702	15,790	38,361
Intervention long working hours	No	76.6	82.5	74.6	66.7	73.9	74.1	79.4	70.0	58.4	68.2
	Yes	23.4	17.5	25.4	33.3	26.1	25.9	20.6	30.0	41.6	31.8
	n(unw)	7,187	8,078	8,982	15,097	39,344	6,966	6,672	8,557	15,493	37,688
Training psychosocial risks	No	80.8	73.4	64.4	45.0	63.1	81.2	73.0	65.0	45.0	62.3
	Yes	19.2	26.6	35.6	55.0	36.9	18.8	27.0	35.0	55.0	37.7
	n(unw)	7,203	8,133	9,040	15,290	39,666	7,070	6,781	8,721	15,848	38,420

A = no WRA; B = WRA without consideration of psychosocial risks; C = WRA considering one aspect of psychosocial risks; D = WRA considering two aspects of psychosocial risks; w = weighted, unw = unweighted; ^a^ only companies with ≥ 20 employees, ^b^ only companies with ≥ 20 employees and companies where employees have to deal with difficult customers, patients, pupils etc

Irrespective of whether a WRA has been carried out or not, “interventions if excessively long or irregular hours are worked” is least common (in 26.1%/31.8% of all companies in 2014//2019) and “procedures to deal with possible cases of threats, abuse or assaults by clients, patients, etc.” is most common (in 55.7%/59.6% of the companies with at least 20 employees in 2014/2019).

Both in 2014 and 2019, the proportion of enterprises confirming PSRM measures is consistently higher among companies that carry out any workplace risk assessment taking psychosocial aspects into account than among those that did not. For example, the percentage of companies with an “action plan stress” is 31.2% (2014) or 36.5% (2019) in companies with WRA-C (considering one out of two aspects of psychosocial risk) and 50.2% (2014) or 56% (2019) in the group of companies with a WRA-D (considering two out of two aspects of psychosocial risk) compared with 13.5% (both 2014 and 2019) for those without any WRA (WRA-A) and 18.4% (2014) or 24.0% (2019) for those without consideration of psychosocial risks in the WRA (WRA-B).

However, even among companies without any WRA, 14–38% (2014) or 14–44% (2019) took specific measures. In both 2014 and 2019, at least a third of the interviewed companies without any WRA confirmed the existence of “procedures to deal with possible cases of threats”, “reorganization of work in order to reduce job demands and work pressure” and “confidential counselling for employees”.

### Association between WRAs and PSRM measures, by survey year

Table 3 shows the predicted percentages (PP) and the average marginal effects (AME) for the association between WRA and PSRM measures in 2014 and 2019, based on logistic regression models, each including company size, industry, sector, country and an interaction term between year of data collection and WRA measure.

Both in 2014 and 2019, the highest PPs are estimated for procedures to deal with threats (from 40%/46% in enterprises without any WRA to 69.8%/73.4% in enterprises with comprehensive WRA-D) and bullying (from 32.5%/36.9% in enterprises without any WRA to 64.5%/69.3% in enterprises with comprehensive WRA-D). The lowest PPs are estimated both in 2014 and 2019 for interventions on long or irregular working hours (from 21.4%/22.3% in enterprises with no WRA to 34.9%/41.3% in enterprises with comprehensive WRA-D).

**Table 3:** Predicted percentages (PP) and AME for the association between comprehensive risk assessment and measures to prevent psychosocial risks by year (pooled ESENER 2 and 3 EU 28 sample; based on separate logistic regression models for each PSRM measure with interaction effect between survey year and respective WRA measure)

	Workplace Risk Assessment														
	2014 (ESENER 2)						2019 (ESENER 3)						Int. effect		n
	WRA-A (PP)	WRA-B (PP)	WRA-C (PP)	WRA-D (PP)	AME (D-A)	p-value	WRA-A (PP)	WRA-A (PP)	WRA-C (PP)	WRA-D (PP)	AME (D-A)	p-value	2nd diff.^d^	p-value	
Action plan stress^a^	13.1	20.3	30.1	50.5	37.3	< 0.001	17.3	24.4	36.0	56.4	39.1	< 0.001	1.8	0.154	39,325
Procedure bullying^a^	32.5	41.0	48.9	64.5	32.0	< 0.001	36.9	46.1	55.8	69.3	32.4	< 0.001	0.4	0.769	39,651
Procedure threats^b^	40.0	44.5	54.3	69.8	29.8	< 0.001	46.0	47.6	60.1	73.4	27.3	< 0.001	-2.5	0.186	24,505
Reorganisation work	34.6	30.7	40.3	50.1	15.5	< 0.001	35.7	33.5	44.7	57.8	22.2	< 0.001	6.7	< 0.001	77,249
Confidential counselling	34.8	31.8	40.2	51.8	17.0	< 0.001	36.3	33.1	43.5	58.0	21.7	< 0.001	4.7	< 0.001	77,190
Conflict resolution procedure	25.0	23.9	30.9	45.4	20.3	< 0.001	26.3	30.2	40.7	55.1	28.8	< 0.001	8.5	< 0.001	77,422
Intervention long working hours	21.4	17.8	26.2	34.9	13.4	< 0.001	22.3	20.3	29.1	41.3	19.0	< 0.001	5.5	< 0.001	76,683
Training psychosocial risk prevention	22.6	27.8	36.7	55.8	33.2	< 0.001	21.3	28.0	37.3	57.6	36.2	< 0.001	3.0	0.001	77,720

All models include the variables company size, industry, sector, country and an interaction between year and respective WRA measure

PP = Predicted percentages; AME = average marginal effects; A = no WRA; B = WRA without consideration of psychosocial risks; C = WRA considering one aspect of psychosocial risks; D = WRA considering two aspects of psychosocial risks

^a^ only companies with ≥ 20 employees

^b^ only companies with ≥ 20 employees and companies where employees have to deal with difficult customers, patients, pupils etc.,

^d^AME 2019 (ESENER-3)– 2014 (ESENER-2)

The PPs for having implemented PSRM measures are consistently higher in companies with a comprehensive WRA (WRA-D) and in companies with WRA considering at least one aspect of psychosocial risk (WRA-C) than in those without a WRA (WRA-A) and in those without considering psychosocial risks in the WRA (WRA-B), both in 2014 and 2019. As shown by the average marginal effects (AMEs), the likelihood of PSRM measures is in 2014/2019 between 13.4%/19% (interventions for long or irregular working hours) and 37.3%/39.1% (action plan to prevent work-related stress) higher in companies that have carried out a comprehensive WRA than in those that have no WRA. Comparatively high AMEs are also documented for “training of employees on how to prevent psychosocial risks such as stress or bullying” (2014: 33.2, 2019: 36.2), for “procedures to deal with possible cases of bullying or harassment” (2014: 32.0, 2019: 32.4) and for a “conflict resolution procedure” (2014: 20.3, 2019: 28.8).

Comparing the AMEs in 2014 and 2019 (see column “2nd diff.” in Table 3), AMEs are higher in magnitude in 2019 than in 2014, with one exception (in case of “procedure threats” a somewhat higher AME was found in 2014 than in 2019). However, the interaction effect corresponding to differences by survey year does not reach statistical significance at the 5% level for “action plan stress” and “procedure bullying”. As these are the measures covered by the restricted sample of enterprises with at least 20 employees, we checked that the significant results for the remaining measures were not simply due to the larger sample size. We therefore conducted an additional sensitivity analysis, where we checked the results for the other variables based on a restricted sample. The interaction effects still were significant for the remaining measures.

However, even among companies without a WRA, the likelihood of having implemented PSRM measures ranged from at least 13.1% (2014) or 17.3% (2019) to max. 40% (2014) or 46% (2019), depending on the measure. The comparatively highest probabilities for measures exist among companies without any WRA for procedures for dealing with possible cases of threats (2014: 40%, 2019: 46%) or bullying (2014: 32.5%, 2019: 36.9%), and for reorganization of work to reduce work demands and pressure (2014: 34.6%, 2019: 35.7%) and confidential counselling for employees (2014: 34.8%, 2019: 36.3%).

### Association between WRAs and PSRM measures, by company size

Table 4 shows the predicted percentages (PP) and the average marginal effects (AME) for the association between comprehensive WRA and PSRM measures differentiated by company size. As these differences do not differ statistically significantly between the years of the study (except for one out of eight measures, results therefore not shown in detail), the regressions investigating differences by company size are not presented for each year of data collection.

**Table 4:** Predicted percentages (PP) and AME for the association between risk assessment and measures to prevent psychosocial hazards by company size (pooled ESENER 2 and 3 EU 28 sample; based on separate logistic regression models for each PSRM measure with interaction effects between company size and respective WRA measure)

	Workplace Risk Assessment														
	Company size ≥ 50						Company size < 50						Int. effect		n
	WRA-A (PP)	WRA-B (PP)	WRA-C (PP)	WRA-D (PP)	AME (D-A)	p-value	WRA-A (PP)	WRA-B (PP)	WRA-C (PP)	WRA-D (PP)	AME (D-A)	p-value	2nd diff.^d^	p-value	
Action plan stress^a^	18.8	25.6	36.3	57.9	39.1	< 0.001	12.8	17.5	27.3	45.7	32.9	< 0.001	6.2	< 0.001	39,325
Procedure bullying^a^	40.8	49.1	57.6	71.5	30.6	< 0.001	30.6	35.7	43.3	58.5	27.9	< 0.001	2.8	0.059	39,651
Procedure threats^b^	48.4	49.2	61.7	74.8	26.4	< 0.001	39.3	41.7	49.8	66.2	26.9	< 0.001	-0.5	0.800	24,505
Reorganisation work	37.1	36.8	47.3	59.6	22.5	< 0.001	34.9	30.1	40.0	50.9	16.0	< 0.001	6.5	< 0.001	77,249
Confidential counselling	44.0	44.6	53.4	66.6	22.6	< 0.001	34.5	27.8	36.0	48.7	14.2	< 0.001	8.4	< 0.001	77,190
Conflict resolution procedure	36.0	36.6	46.0	62.0	26.0	< 0.001	24.3	23.0	30.5	43.9	19.6	< 0.001	6.4	< 0.001	77,422
Intervention long working hours	29.1	26.1	34.8	45.9	16.8	< 0.001	21.0	16.3	24.1	34.0	13.0	< 0.001	3.8	0.003	76,683
Training psychosocial risk prevention	26.2	33.4	42.7	62.9	36.6	< 0.001	21.4	25.8	34.1	53.3	31.9	< 0.001	4.8	< 0.001	77,720

All models include industry, sector, country, survey year and an interaction between company size and respective WRA measure

PP = Predicted percentages; AME = average marginal effects; A = no WRA; B = WRA without consideration of psychosocial risks; C = WRA considering one aspect of psychosocial risks; D = WRA considering two aspects of psychosocial risks

^a^ only companies with ≥ 20 employees, ^b^ only companies with ≥ 20 employees and companies where employees have to deal with difficult customers, patients, pupils etc.; ^d^AME ≥ 50– AME < 50

As shown by the AMEs in Table 4, both small (< 50 employees) and larger companies (≥ 50 employees) are more likely to adopt PSRM measures when a comprehensive WRA is in place. In both groups, the AMEs are largest in magnitude for the presence of an “action plan to prevent work-related stress” (39.1 vs. 32.9), “employee training to prevent psychosocial risks” (36.6 vs. 31.9) and procedures to deal with “possible cases of bullying” (30.6 vs. 27.9) or “threats from customers, patients, etc.” (26.4 vs. 26.9). The associations are weakest (corresponding to smaller AME) in case of “interventions if long or irregular hours are worked” (16.8 vs. 13.0), “reorganization of work in order to reduce job demands and work pressure” (22.5 vs. 16.0) and “confidential counselling for employees” (22.6 vs. 14.2).

However, as shown by the differences in AME values (see column “2nd diff.” in Table 4), the associations between WRA and PSRM measures are in most cases lower in small companies than in larger ones. The exceptions are the “procedures for dealing with possible cases of threats, abuse, or assault by customers” and for “dealing with possible cases of bullying or harassment”, where the differences in AME by company size did not reach statistical significance. The highest differences in AME are found for “confidential counselling for employees” (2nd diff.=8.4), “reorganization of work in order to reduce job demands and work pressure” (2nd diff.=6.5), “conflict resolution procedure” (2nd diff.=6.4) and the existence of an “action plan to prevent work-related stress” (2nd diff.=6.2). Therefore, the results indicate that the strength of the association between the measures and the presence of comprehensive WRA is smaller in small enterprises than in larger ones.

## Discussion

### Significance of the results

Companies that carry out WRAs including psychosocial risks are more likely to implement PSRM measures than those not including psychosocial risks or those without WRAs at all. This is true for small enterprises as well as for larger establishments. These results are in line with existing evidence that a good OSH organization in the company, including the implementation of comprehensive WRAs, is an important conducive condition for specific PSRM measures (European Agency for Safety and Health at Work 2012; Leka and Cox 2008; Dahler-Larsen et al. 2020). The present study thus underlines the importance of current efforts at national and European level to work towards improving the quality of OSH structures and processes in companies and towards more widespread implementation of comprehensive WRAs (European Foundation for the Improvement of Living and Working Conditions and European Agency for Safety and Health at Work 2014; Walters and Wadsworth 2016; Pavlista et al. 2021). Our study points out that the association between WRA and a majority of the PSRM measures has become stronger in 2019 than in 2014. This supports the assumption that European and national efforts made in recent years (e.g. in the framework of the EU Strategic Framework on Health and Safety at Work 2014–2020 and via the EU OSHA campaign on psychosocial risks and stress at work) are effective to improve the consideration of psychosocial risks in companies’ OSH policies and WRAs.

However, even among companies that do not implement a WRA, we observed a considerable proportion of companies that still implemented PSRM measures, especially among small companies. Therefore, this study also adds to the current knowledge, which has so far been limited to national surveys and has not considered the role of company size. For example, data from the Netherlands show that up to 37% of companies without WRAs (and 45% of the companies whose WRAs do not take “work stress” into account) have nevertheless taken measures to reduce work stress (Inspectie SZW 2016). Similarly, the French working conditions survey of 2016 found that the prevalence of preventive measures targeting psychosocial risks exceeded that of documented psychosocial risk assessments (34% vs. 15%) (Amira and Desjonquères 2017). These findings suggest that considering only the existence of a (comprehensive) WRA leads to an underestimation of the proportion of establishments that actually implement PSRM measures. This is particularly the case for small enterprises, where, according to the analyses presented here, the association between a WRA and the implementation of PSRM measures is less pronounced than for larger companies. Although very little research has been done on this topic (Pinder et al. 2016), it is possible that small companies are more likely to rely on informal and implicit (but not necessarily ineffective) ad hoc practices of “assessing” and “managing” occupational risks (Beck et al. 2017; Lenhardt 2017; European Agency for Safety and Health at Work 2019c). Our study results support the hypothesis that PSRM also takes place independently of the implementation of formal WRA and thus also beyond the structures and processes of OSH in companies, e.g., in the context of human resource management, employee-oriented leadership, or as a component of professional occupational practice of the employees (Beck et al. 2017). This implies that the management of work-related psychosocial risks can and should be addressed and (further) developed not only in the context of OSH, but also in other operational contexts (Rothe et al. 2017). Such practices may not be detected if respondents are only asked about the implementation of formal WRA procedures.

### Strengths and limitations of the study

This study examines the relationship between WRAs and PSRM measures based on a very large sample of enterprises from 28 EU countries (as of 2019), The ESENER-2 and − 3 data used here allow estimating the likelihood of PSRM measures in the presence or absence of (comprehensive) WRAs, differentiated for small and large companies. However, there are several limitations to consider:

This study is based on cross-sectional data that do not allow any conclusions to be drawn about cause-effect relationships between the variables examined here. It should also be noted that the ESENER does not provide information on whether and to what extent the PSRM measures were actually developed and implemented in the context of a WRA. It is quite possible, and even likely, that even for companies that conduct WRAs including psychosocial risks, at least some of the PSRM measures were taken independently of the WRA (see discussion above). Thus, the association between WRA and PSRM measures reported here is no more and no less than a measure of the simultaneous presence of these aspects in companies. However, if PSRM measures are implemented in enterprises where there is no WRA at all, it can be concluded that these measures have been taken independently of workplace risk assessments. Another limitation is that some of the PSRM measures (action plan stress, procedure bullying, procedure threats) were only assessed in companies with 20 or more employees (as pre-tests suggested that these questions were not relevant for smaller companies). As a result, for example, there is no information on bullying and threatening behaviour for small businesses– even if such behaviour exists. We therefore may have missed this information. One example are home care services, which often have less than 20 employees, but still can have problems with aggressive clients.

Another disadvantage of the ESENER study is that the PSRM variables are rather crude and do not give a precise indication of which specific measures have been implemented, whether they are organisational or behavioural interventions. Therefore, the results of the correlation between WRA and measures as determined in this study cannot be generalised. They may be different depending on the type of measure.

The PPs and AMEs estimated here are adjusted for EU country, industry and sector. However, the possible effect-modifying influence of these variables has not been investigated here in a differentiated way. In particular, it is conceivable that the national regulatory context of OSH and PSRM could have an impact on the results. Although the EU Framework Directive (Council of the European Communities 1989) provides a binding framework for OSH and the consideration of psychosocial risks in all EU countries, there are, however, considerable differences between the EU countries in the concrete design (European Commission 2014; Leka et al. 2015). Available studies suggest that national stress legislations are associated with more companies having specific measures to prevent psychosocial risks (European Agency for Safety and Health at Work 2019c; Jain et al. 2022). In this sense, it can be assumed that the association between WRA and PSRM is more pronounced in EU countries where there are explicit and strictly controlled OSH regulations for the implementation of PSRM measures than in EU countries where this is less the case.

It should also be noted that ESENER suffers from low response rates (ESENER-2: 24%, ESENER-3: 18%, see European Agency for Safety and Health at Work 2020, p.61f), which may have led to selection bias. Yet, it can be assumed that companies that do not invest much in OSH and PSRM are less likely to participate in the survey. There is therefore a high probability that the prevalence of WRA and PSRM measures presented here is even overestimated. There may also be a classification bias due to the indirect measurement of the implementation of comprehensive WRAs in ESENER. As the consideration of “supervisor-employee relationships” and/or “organisational aspects such as work schedules, breaks or work shifts” in WRAs are used as proxy indicators, the requirements to be classified as having implemented a comprehensive WRA including psychosocial risks are comparatively low. More detailed studies are required to draw far-reaching conclusions at this point where the assessment of WRAs needs to be refined, by including additional details on procedures and aspects covered as part of the WRA. On a more general note, these limitations also point to possible extensions of our study, including studies that explicitly address health-related consequences of WRAs and PSRM measures. In addition, qualitative studies appear instructive at this point to complement our study, with different strategies to address selection and classification biases (Morse et al. 2002).

## Conclusions

The findings underline the importance of a WRA that includes psychosocial risks as a means of implementing PSRM measures, but also advocates for a broader perspective that considers measures taken independently of legal occupational safety and health (OSH) standards, especially in small enterprises. In which contexts, to what extent and under what conditions PSRM measures are implemented in companies should be explored in further research.

The implementation of (comprehensive) WRA and PSRM measures should be distinguished and studied as two different aspects of PSRM in future research. These two aspects are related, as our study shows, in line with other studies. Further research should explore the possible determinants of this association. As discussed above, the possible role of national OSH regulations on the implementation of PSRM measures should be further investigated.

## Data Availability

European Agency for Safety and Health at Work, TNS Infratest Sozialforschung (Munich) (2016) Second European Survey of Enterprises on New and Emerging Risks, 2014. [data collection]. 2nd Edition. UK Data Service. SN: 7808. 10.5255/UKDA-SN-7808-1. Irastorza X, Cavet M, Cockburn W, Riedmann A, Strauss A, Houtman I, Vanadziņš I (2020) Third European Survey of Enterprises on New and Emerging Risks 2019 (ESENER-3). GESIS Datenarchiv, Köln. ZA7735 Datenfile Version 1.0.0. 10.4232/1.13649.
